# Psychometric assessment of the US person-centered prenatal and maternity care scales in a low-income predominantly Latinx population in California

**DOI:** 10.1186/s12905-023-02721-5

**Published:** 2023-11-17

**Authors:** Patience A. Afulani, Kimberly Coleman-Phox, Daisy Leon-Martinez, Kathy Z. Fung, Erica Martinez, Mary A. Garza, Charles E. McCulloch, Miriam Kuppermann

**Affiliations:** 1 Department of Epidemiology & Biostatistics, University of California, San Francisco, USA; 2 Department of Obstetrics, Gynecology, & Reproductive Sciences, University of California, San Francisco, USA; 3 Central Valley Health Policy Institute, College of Health and Human Services, California State University, Fresno, USA; 4 Department of Public Health, College of Health and Human Services, California State University, Fresno, USA

**Keywords:** Person-centered care, Prenatal care, Intrapartum care, Quality of care, Latinx, Low-income

## Abstract

**Objectives:**

To assess psychometric properties of two scales developed to measure the quality of person-centered care during pregnancy and childbirth in the United States—the Person-Centered Prenatal Care (PCPC-US) and Person-Centered Maternity Care (PCMC-US) scales—in a low-income predominantly Latinx population in California.

**Methods:**

Data were collected from July 2020 to June 2023 from surveys of low-income pregnant and birthing people in Fresno, California, participating in the “Engaging Mothers and Babies; Reimagining Antenatal Care for Everyone” (EMBRACE) trial. Research staff administered the 26-item PCPC-US scale at 30–34 weeks’ gestation (*n* = 315) and the 35-item PCMC-US scale at 10–14 weeks after birth (*n* = 286), using the language preferred by the participant (English or Spanish). We assessed construct, criterion, and known group validity and internal consistency of the scales.

**Results:**

78% of respondents identified as Latinx. Factor analysis identified one dominant factor for each scale that accounted for over 60% of the cumulative variance, with most items loading at > 0.3. The items also loaded adequately on sub-scales for “dignity and respect,” “communication and autonomy,” and “responsive and supportive care.” Cronbach’s alpha for the full scales were > 0.9 and between 0.70 and 0.87 for the sub-scales. Summative scores range from 0 to 100, with higher scores indicating higher person-centered care. Correlations with scores on scales measuring prenatal care quality and birth experience provided evidence for criterion validity, while associations with known predictors provided evidence for known-group validity.

**Conclusions:**

The PCPC-US and PCMC-US scales, which were developed using a community-engaged process and found to have good psychometric properties in a largely high-income sample of Black women, were shown to also have good psychometric properties in a sample of low-income primarily Latinx women. Both scales provide valid and reliable tools to measure person-centered care experiences among minoritized communities to support efforts to reduce existing birth inequities.

**Supplementary Information:**

The online version contains supplementary material available at 10.1186/s12905-023-02721-5.

## Introduction

Inequities in maternal and neonatal outcomes in the US are deeply rooted and disproportionately affect Black, Indigenous, and other racial and ethnic minoritized women and gender expansive pregnant and birthing people (subsequently referred to as women or pregnant or birthing people for brevity). Black women experience maternal mortality [1, 2], severe maternal morbidity [3], and preterm birth [4, 5] at rates 2.6, 2.0, and 1.5 times higher, respectively, than White women. Prior to the COVID-19 pandemic, Latinx women had lower maternal mortality rates than White women; however, in 2021 Latinx maternal death rates exceeded rates for White birthing people (28 vs 26.6 deaths per 100,000 live births) [2]. Latinx women also experience higher rates of severe maternal morbidity and preterm birth compared to White women [4, 6, 7]. Differences in educational attainment and clinical risk factors do not explain the disparate rates [8, 9], and a growing literature points to underlying social and structural determinants of adverse birth outcomes including racism and classism [10–12]. Rates of severe maternal morbidity and mortality are widely used indicators of quality of care during pregnancy, childbirth, and postpartum, but they reveal little about the person-centeredness of care [13].

Person-centered care—care that is respectful and responsive to people’s needs, preferences, and values—is a key dimension of quality [14], which is often lacking in the care encounters of minoritized groups, who describe their prenatal and childbirth healthcare experiences as disrespectful and disempowering due to information being withheld and being dismissed [15–17]. In population-based surveys, Black, Latinx, and individuals who spoke a primary language other than English were more likely to report unfair treatment based on these characteristics during their childbirth hospital stay compared to White and English-speaking individuals [18, 19]. In another large US study, Black, Latinx, and Indigenous women were most likely to report mistreatment [20]. Among Latinx individuals, along with socioeconomic status (SES), insurance, and language, qualitative studies reveal that immigration status may influence experience of care [17, 21–24]. Negative interactions with the healthcare system are implicated in decreased access, utilization, and adverse birth outcomes [25, 26]. Given that nearly 24% of US births and 46% of California births are to Latinx-identifying people, more nuanced information about their maternity care experience is critical to providing high-quality equitable care [27, 28].

Despite a large body of evidence demonstrating disparities in care among minoritized pregnant and birthing people, few tools to comprehensively measure the extent of person-centered care during pregnancy and birth have been specifically validated in these populations. Although there are several scales for evaluating patient and family experience in Latinx populations, none have been validated in both English- and Spanish- speaking US populations to holistically measure quality of person-centered prenatal and intrapartum care [29–32]. To address this gap, a rigorous and community-engaged scale development process was used to create the US versions of the person-centered prenatal care (PCPC-US) and the person-centered maternity care (PCMC-US) scales for prenatal and intrapartum care, respectively [33, 34]. This process involved an initial review to build on items in the PCMC scale developed and validated in Africa and Asia [35–37], followed by expert reviews, cognitive interviews, pretesting, survey administration, and psychometric analysis—with decisions at each step guided by a community advisory board (CAB) made up of women from racial and ethnic minoritized groups [33, 34]. Both scales have three subscales measuring “dignity and respect”, “communication and autonomy”, and “responsive and supportive care”;, and both have high content validity [33, 34]. Although the expert review and cognitive interviews conducted in the process of developing the items for the PCPC-US and PCMC-US scales included Latinx women, the final validation sample was made up of predominantly Black women with relatively high-income and education [33, 34]. Both scales had high construct and criterion validity and as well as high internal consistency reliability in this predominantly high SES Black sample, with Cronbach alphas of > 0.9 for the main scales and > 0.8 for the subscales [33, 34]. In this paper we seek to assess the psychometric properties of the PCPC-US and PCMC-US scales in a low SES predominantly Latinx population.

## Methods

### Participants and procedures

Data for this analysis are from an ongoing randomized controlled trial entitled “Engaging Mothers & Babies – Reimagining Antenatal Care for Everyone” (EMBRACE) [38]. The EMBRACE study seeks to assess the comparative effectiveness of two forms of enhanced prenatal care among low-income pregnant and birthing people in Fresno, California. Participants were recruited from several clinical sites in Fresno. The data for this analysis were collected between July 2020 and June 2022. The recruitment and study procedures have been previously described [39] and are summarized here. Research staff worked closely with clinic staff to identify potentially eligible participants by reviewing appointment records and approaching them in-person in the waiting rooms of participating clinical sites or remotely over the telephone. Inclusion criteria were: 1) < 25 weeks gestation (with pregnancy confirmed by ultrasound if < 8 weeks), 2) eligible for Medicaid (i.e., ≤ 213% of the federal poverty level) and 3) able to speak English or Spanish. Exclusion criteria were: 1) not planning to continue prenatal care with the participating provider, 2) not able to legally consent to study participation, or 3) not available to attend group prenatal sessions at the scheduled times. Eligible and interested individuals signed the informed consent form and completed an interviewer-administered baseline questionnaire. Participants were then allocated to group or enhanced individual prenatal care per the randomization schedule. Subsequently, participants completed a third trimester questionnaire (between 30 and 34 weeks gestation) and a postpartum questionnaire (between 10 and 14 weeks after the delivery) administered over the telephone or in person by a research team member. Participants received $30 remuneration for completing the baseline questionnaire and $50 for the third trimester and postpartum questionnaires they completed. The study was approved by the University of California, San Francisco, and California State University, Fresno, Institutional Review Boards.

### Measures

The dependent variables for this paper are person-centered care during pregnancy and birth measured with the 26-item PCPC-US [33] and the 35-item PCMC-US scales [34], respectively. To establish equivalence in meaning between the English and Spanish versions of the scales during the initial development, the scale items were translated and back translated by a certified translation agency and reviewed by a team member fluent in Spanish following expert reviews. Cognitive interviews were then conducted in both English and Spanish, and all versions were updated following cognitive interviews and finalized as self-administered surveys. For the EMBRACE study, the self-administered English scales were edited by the EMBRACE study PI (MK) for use as interviewer-administered surveys and reviewed by the original validation study PI (PAA) and EMBRACE study field staff. The self-administered Spanish versions were then edited by a team member fluent in Spanish (EM), to reflect edits made to the English versions, and reviewed and back translated by a certified translation agency. A team member (EM) and field staff fluent in Spanish performed a final review before pretesting the scales with potential participants and administration in the EMBRACE study. Wording of all scale questions (English version) are shown in Table 1. Items on both scales have the following options with the noted scoring: No, never (0); Yes, a few times (1); Yes, most of the time (2); and Yes, all the time (3). A few questions have a “not applicable” option. Scores on both scales are generated by adding scores of the individual items (after reverse coding negatively worded items and recoding not applicable options to the upper middle category such that all item responses range from 0 to 3, with higher numbers indicating the desired behaviors). The summative score is then standardized to range from 0–100 with higher scores indicating the receipt of more person-centered care.

**Table 1:** Items in the scales

*No*	*Question*	*Label used in text*
	**26 items in the Person-centered Prenatal Care United States (PCPC-US) Scale**	
	Did your providers …	
1	Introduce themselves to you when they first saw you?	Introduction
2	Knock on the door and wait for a response before entering?	Privacy-knock
3	Treat you with respect?	Treat you with respect
4	Respect your family or companions who were with you?	Respect your family
5	Involve you in decisions about your care?	Involved in decisions
6	Explain to you why they were doing examinations or procedures on you?	Explain procedures
7	Ask your permission/consent before touching you or examining you or starting a procedure you?	Consent
8	Cover or screen you during exams so that you did not feel exposed?	Privacy-not exposed
9	Encourage you to ask questions?	Encourage you to ask questions
10	Check that you understood information that was given to you?	Check you understood information
11	Give you information in a way that showed they cared about you?	Information showed they cared
12	Ask about your emotional well-being?	Emotional well-being
13	Provide you with resources to help with your emotional well-being if you needed it?	Resources for emotional wellbeing
	Did you feel your providers …	
14	Avoided, ignored, or otherwise neglected you?	Neglected
15	Took the best care of you?	Best care
16	Insulted, threatened, or talked to you rudely?	Verbal abuse
	[Note to interviewer: “Talking rudely” would include “shouting at you” and “scolded you.”]	
17	Handled you roughly, held you down, or physically restrained you?	Physical abuse
	Did you feel …	
18	Heard and listened to by your providers?	Heard and listened to
19	Your health information was kept confidential and private by providers and staff?	Information confidentiality
20	You could ask your providers any questions you had?	Could ask any questions
21	Your questions were answered when you asked them?	Questions were answered
22	Your experience and knowledge were valued?	Knowledge valued
23	You could completely trust your providers with regards to your care?	Trust
24	Physically safe in the place you received prenatal care?	Safe
25	In general, how did you feel about the amount of time the providers spent with you?	Time with provider
26	Would you say you were discriminated against because of your race, ethnicity, sexual orientation, immigration status or anything else?	Discrimination
	**35 items in the Person-Centered Maternity Care, United States (PCMC-US) Scale**	
	Did your providers …	
1	Introduce themselves to you when they first saw you?	Introduction
2	Treat you with respect?	Treat you with respect
3	Respect your family or companions who were with you?	Family respected
4	Speak to you using language or words you could understand?	Language understood
5	Ask your permission/consent before touching you or examining you or doing procedures on you?	Consent
6	Involve you in decisions about your care?	Involved in decisions
7	Explain to you why they were doing examinations or procedures on you?	Explain procedures
8	Explain to you why they were doing examinations or procedures on your baby?	Explain baby procedures
9	Check that you understood information that was given to you?	Checked understanding
10	Cover or screen you during exams so that you did not feel exposed?	Privacy-covered
11	Ask about your emotional well-being?	Emotional well-being
	Did you feel your providers …	
12	Respected your customs and culture?	Customs respected
13	Avoided, ignored, or otherwise neglected you?	Neglected
14	Responded in a timely manner when you requested assistance?	Timely response
15	Believed you when you said you were in pain?	Believed about pain
16	Did everything they could to help you manage your pain?	Pain management
17	Took the best care of you?	Took best care
18	Insulted, threatened, or talked to you rudely?	Verbal abuse
19	Handled you roughly, held you down, or physically restrained you?	Physical abuse
20	Respected your feeding choice for your baby?	Baby feeding choice respected
	Did you feel …	
21	Heard and listened to by your providers?	Felt heard
22	Informed about what was happening to you during your childbirth?	Felt informed
23	Coerced or pressured into a decision by your providers?	Coercion
24	Your birth plan or preferences were respected?	Birth preference respected
25	Your health information was kept confidential and private by providers and staff?	Information confidential
26	You could ask your providers any questions you had?	Could ask questions
27	Your experience and knowledge were valued?	Experience valued
28	You could completely trust your providers with regards to your care?	Trust
29	Physically safe in the place you gave birth?	Felt safe
30	How did you feel about the amount of time you had to wait before being examined by a health care provider?	Wait time
31	Were you able to give birth in the position of your choice?	Birth position of choice
32	Were you allowed to have everyone you wanted to be with you during your childbirth?	Companionship
33	Did you receive the support you needed to reach your feeding goals for your baby?	Support for baby feeding
34	Were you supported in creating a birth environment that made you feel comfortable?	Comfortable birth environment
35	Would you say you were discriminated against because of your race, ethnicity, sexual orientation, immigration status or anything else?	Discrimination

Abbreviations: *PCPC-US* person-centered prenatal care, *PCMC-US* person-centered maternity care

Because there are no gold standard measures for person-centered prenatal and intrapartum care, we included two other instruments designed to measure experience of care during pregnancy and birth—the Prenatal Care Satisfaction Questionnaire (PSQ) [40] and the Mothers on Respect index (MORI) [41]—to assess criterion validity of the PCPC-US and PCMC-US scales. These two scales are used because they measure constructs related to person-centered care. The PSQ is a 22-item scale that measures five dimensions of prenatal care quality including art of care, technical quality, physical environment, access, availability, and overall satisfaction with prenatal care. It was validated in a sample of low-income African American and Mexican American mothers (74% African American and 25% Mexican American) and shown to have high internal consistency in that sample (Cronbach’s alpha of 0.95) [40]. MORI is a 14-item scale capturing patient experiences and interactions with providers during prenatal and postnatal care, with a focus on the patient’s ability to exercise autonomy without discrimination. It was validated in samples of women in Canada and the US (58% Caucasian, 11% Black, 8% Hispanic/Latina, 19% Asian, African, other or biracial) and shown to have high internal consistency (Cronbach’s alpha of 0.94) [41]. The PSQ was administered during the third trimester and MORI was administered during the third trimester and postpartum.

We included participant-reported sociodemographic characteristics, measures of socioeconomic status, health-related factors, and other variables associated with care experience to describe the sample and assess known-group validity (Table 2) [42, 43]. Sociodemographic characteristics include age, parity, marital status, birth country, and race and ethnicity. Race and ethnicity were included as proxies for exposure to structural and institutional racism and not as biological variables and measured by asking participants to indicate the category with which they most identified. Measures of socioeconomic status included monthly household income before taxes, employment status, highest educational attainment, insurance status, and housing instability (ever been homeless). For health-related factors, we included history of diabetes (Type I, Type II, or gestational) or hypertension (hypertension, high blood pressure, or preeclampsia); history of mental health disease or disorders; history of preterm birth (< 37 weeks’ gestation), low birth weight (< 5.5 lbs or 2,500 g), or other complication (bleeding or threatened miscarriage in current pregnancy); history of ever testing positive for COVID; and self-rated health. We also measured frequency and worry about discrimination (see Table 2). These variables were all measured at baseline, except self-rated health, which was measured during the third trimester, and so only included as a predictor for the PCMC-US analysis. Thus, all the predictors preceded the outcomes. We also included the screening and delivery period (coded as COVID-19 pre-vaccine, mass vaccination, and medication availability periods) to capture contextual factors related to the evolving COVID-19 pandemic. Measurement of the variables has been previously described [39]. All questions were pretested with participants prior to being administered in the survey.

**Table 2:** Baseline characteristics of study participants

Characteristics ^a^	PCPC-US subsample, *n* = 315	PCMC-US subsample, *n* = 286
Age, years		
< 18	7 (2.2)	4 (1.4)
18–24	101 (32.1)	99 (34.6)
25–34	165 (52.4)	149 (52.1)
≥ 35	38 (12.1)	30 (10.5)
Missing	4 (1.3)	4 (1.4)
Relationship status		
Married, living with partner	251 (79.7)	225 (78.7)
Partnered, not living together	35 (11.1)	35 (12.2)
Single	29 (9.2)	26 (9.1)
Parity		
0	81 (25.7)	78 (27.3)
1	84 (26.7)	72 (25.2)
2	67 (21.3)	63 (22.0)
3	50 (15.9)	44 (15.4)
4 +	32 (10.2)	28 (9.8)
Missing	1 (0.3)	1 (0.4)
Race or ethnic group		
African American or Black	15 (4.8)	13 (4.6)
Asian or Pacific Islander	11 (3.5)	9 (3.2)
Bi- or multi-racial/ethnic	10 (3.2)	10 (3.5)
Latina, Latinx, or Hispanic	246 (78.1)	226 (79.0)
None of the above ^b^	4 (1.3)	3 (1.1)
White	29 (9.2)	25 (8.7)
Birth country		
United States	200 (63.5)	183 (64.0)
Mexico	101 (32.1)	91 (31.8)
El Salvador	3 (1.0)	2 (0.7)
Guatemala	3 (1.0)	3 (1.1)
Honduras	1 (0.3)	1 (0.4)
Nicaragua	1 (0.3)	1 (0.4)
Philippines	2 (0.6)	2 (0.7)
Armenia	1 (0.3)	1 (0.4)
Egypt	1 (0.3)	1 (0.4)
India	1 (0.3)	1 (0.4)
Missing	1 (0.3)	1 (0.4)
Interview language – Spanish	82 (26.0)	75 (26.2)
Highest level of education attained		
Less than high school diploma, high school graduate, or GED	181 (57.5)	161 (56.3)
Some college	101 (32.1)	96 (33.6)
College graduate or professional or graduate degree	32 (10.2)	28 (9.8)
Missing	1 (0.3)	1 (0.4)
Monthly household income, $		
< 1000	64 (20.3)	54 (18.9)
1000 – 2000	103 (32.7)	98 (34.3)
2001 – 3000	76 (24.1)	73 (25.5)
> 3000	58 (18.4)	49 (17.1)
Missing	14 (4.4)	12 (4.2)
Currently Employed		
No	204 (64.8)	182 (63.6)
Yes	111 (35.2)	103 (36.0)
Missing	0 (0.0)	1 (0.4)
Medi-Cal Coverage		
No	19 (6.0)	18 (6.3)
Yes	296 (94.0)	268 (93.7)
Ever been homeless		
No	267 (84.8)	240 (83.9)
Yes	42 (13.3)	39 (13.6)
Missing	6 (1.9)	7 (2.5)
Diagnosis of diabetes or hypertension	67 (21.3)	57 (19.9)
Diagnosis of mental health condition	75 (23.8)	64 (22.4)
Prior preterm birth, low birth weight infant, or other pregnancy complication	82 (26.0)	79 (27.6)
How often do you feel discriminated against because of your race or ethnicity?		
Never	181 (57.5)	167 (58.4)
Rarely	59 (18.7)	54 (18.9)
Sometimes	63 (20.0)	56 (19.6)
Often	12 (3.8)	9 (3.2)
How often do you worry that you are treated or judged unfairly because of your race or ethnicity?		
Never	164 (52.1)	152 (53.2)
Not very often	67 (21.3)	59 (20.6)
Somewhat often	65 (20.6)	57 (19.9)
Very often	19 (6.0)	18 (6.3)
Received any care by phone or video		
No	218 (69.2)	169 (59.1)
Yes	97 (30.8)	90 (31.5)
Missing	0 (0.0)	27 (9.4)
Have you ever tested positive for COVID-19?		
No	177 (56.2)	168 (58.7)
Yes	88 (28.0)	67 (23.4)
Not asked	50 (15.9)	51 (17.8)
How do you describe your general health?		
Excellent	66 (21.0)	72 (25.2)
Very good	64 (20.3)	69 (24.1)
Good	94 (29.8)	103 (36.0)
Fair or poor	34 (10.8)	41 (14.3)
Missing	57 (18.1)	1 (0.4)
Screening Period		
COVID-19 Pre-Vaccine^c^	81 (25.7)	84 (29.4)
COVID-19 Mass-Vaccine^d^	114 (36.2)	128 (44.8)
COVID-19 Medication^e^	120 (38.1)	74 (25.9)
Delivery Period		
COVID-19 Pre-Vaccine^c^	11 (3.5)	12 (4.2)
COVID-19 Mass-Vaccine^d^	119 (37.8)	129 (45.1)
COVID-19 Medication^e^	148 (47.0)	145 (50.7)
No Delivery data	37 (11.8)	

*Abbreviations: PCPC-US* person-centered prenatal care, *PCMC-US* person-centered maternity care, *GED* general education development certificate, *COVID-19* Coronavirus disease -19

^a^Data are reported as No. (%) of participants unless otherwise indicated

^b^Data include Native American, American Indian, and Alaska Native

^c^COVID-19 Pre-Vaccine dates include 03/16/20 – 04/30/21

^d^COVID-19 Mass-Vaccine dates include 05/01/21 – 02/28/22

^e^COVID-19 Medication dates include 03/01/22 – present

### Analysis

The current analysis uses baseline sociodemographic data collected pre-randomization, prenatal experience data collected in the third trimester, and birth experience data collected in the postpartum interview from July 2020-June 2023. There are thus two analytic samples for the primary outcomes. The sample for the PCPC-US analysis consists of 315 individuals and for PCMC-US consists of 286 people (*n* = 29 difference due to some participants who were interviewed during the third trimester having yet to complete their postpartum interview). We first conducted univariate analysis to characterize the sample. We used descriptive statistics to examine frequency and percentage of categorical variables and means and standard deviations of continuous variables.

Content validity of the scales was assessed during their initial development through expert reviews and cognitive interviews [33, 34]. In this study, we focus on construct validity—the extent to which items represent the underlying construct; criterion validity—the extent to which scale scores are correlated with related measures; and internal consistency reliability [44, 45]. We assessed construct validity using inter-item correlation and exploratory factor analysis. Average inter-item correlations of between 0.20 and 0.40 are said to be within the ideal range, while factor loadings of > 0.3 are the generally used cut-off for factor loadings [46]. We used the Kaiser–Meyer–Olkin (KMO) measure of sampling adequacy to assess suitability of the variables for factor analysis, Kaiser’s rule of retaining only factors with eigenvalues exceeding unity and the “break” in the scree plot to determine number of factors, and factor loadings and uniqueness to assess performance of individual items. Oblique rotations, which allow for correlation between the rotated factors, was used given person-centered care domains are theoretically related [47]. Internal consistency reliability was assessed using Cronbach’s alpha: values ≥ 0.7 are generally considered acceptable evidence of reliability [44, 45, 47].

We generated summative scores using all the scale items and the items included in the sub-scales. Scores on each scale were then standardized by dividing the score by the maximum possible (e.g., 78 (26*3) for the 26-item PCPC-US scale and 105 (35*3) for the 35-item PCMC-US scale) and then multiplying by 100. This creates a standardized score that ranges from 0–100 for all, where increasing scores reflect more person-centered care. As a means of assessing criterion validity, we hypothesized that the PCPC-US and PCMC-US scales scores would be strongly correlated with related measures of experience of prenatal and intrapartum care, respectively [33–35]. We thus examined correlations between scores on the PCPC-US scale and both the PSQ and MORI scales measured during the third trimester and correlations between PCMC-US and MORi scores on birth experience measured postpartum.

We also examined patient factors associated with the PCPC-US and PCMC-US scale scores to assess known-groups validity—i.e., whether a tool can discriminate between groups known to differ on the variable of interest [42, 43]. For this, we first conducted bivariate analyses using crosstabulations of means and unadjusted ordinary least squares regressions. All variables that had *p*-values of < 0.05 in the bivariate analysis were then included in an initial saturated multivariate model and backwards stepwise linear regression used to select variables to include in the final multivariate model. Given that the score distributions for both scales were left skewed, we ran the final model using bootstrapping with 1000 replications [48]. Analyses were conducted using Stata 16 [49].

## Results

### Participants demographics

Baseline characteristics of participants for the PCPC-US and PCMC-US samples are presented in Table 2. Most participants were between 18 and 35 years (85% and 87% for PCPC-US and PCMC-US samples, respectively) and married (80%, 89%) and about a quarter were nulliparous. Most also identified as Latina, Latinx, or Hispanic (78%, 79%), were born in the US (63%, 64%), and completed the interviews in English (74%, 74%). More than half (58%, 56%) had only high school education and about two out of three were not currently employed. The average monthly household income for most was less than $3000 and 13% reported having been homeless at some point. Although all participants were eligible for Medi-Cal, 94% reported having Medi-Cal coverage.

### Psychometric analysis

Distributions for the individual PCPC-US and PCMC-US items are in Additional file 1: Appendix 1a and 2a and score distributions in Additional file 1: Appendix 1b and 2b respectively.

#### PCPC-US scale

**Fig. 1:**
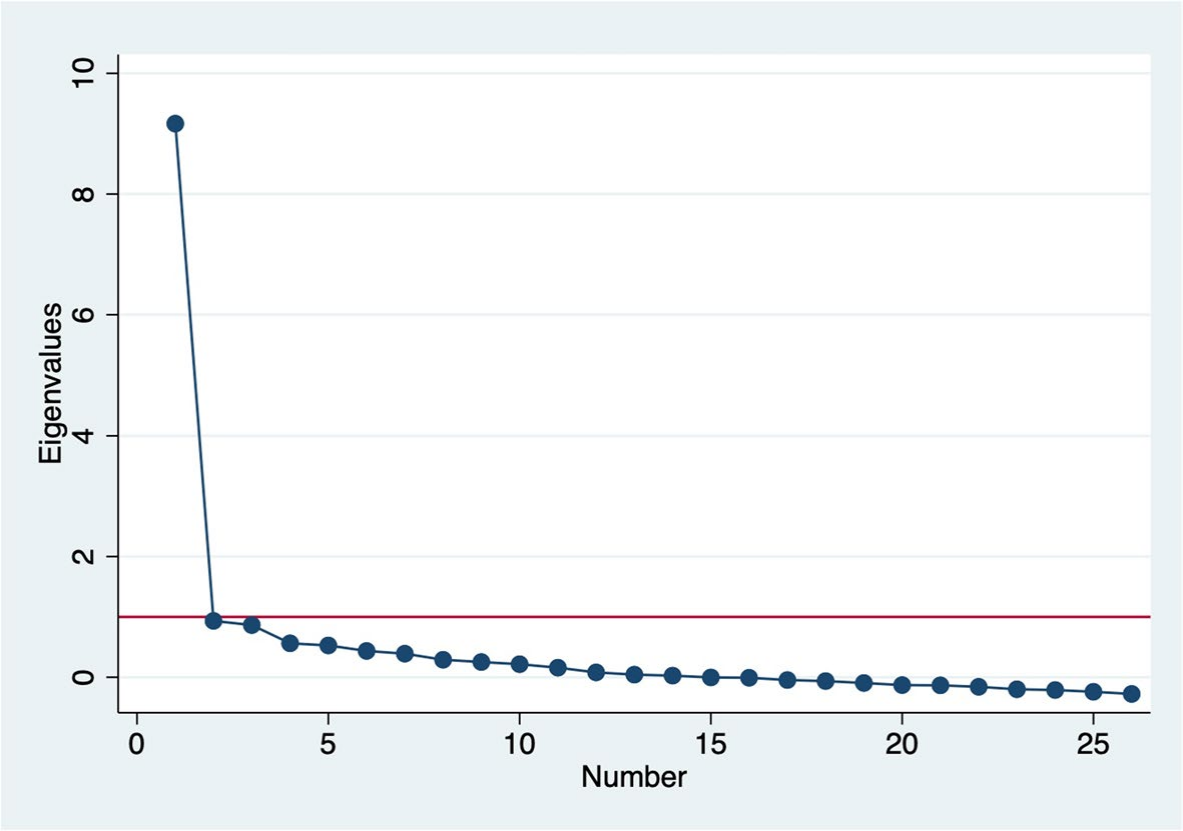
Scree plot from factor analysis of 26 person-centered prenatal care-US scale items

In general, there was good correlation between most items in the PCPC scale. The average inter-item correlation was 0.31, with most inter-item correlations between 0.2 and 0.7. Six items (introductions, family respected, information confidentiality, knocked on door, covered to respect privacy, and physical abuse) had inter-item correlations of less than 0.3 with most other items. The KMO values ranged from 0.41–0.93, with an overall KMO of 0.89, indicating that the variables were satisfactory for factor analysis [50]. Factor analysis of the 26 PCPC-US items yielded one dominant factor (Fig. 1) with an eigenvalue of 9.16 accounting for 74% of the cumulative variance. All items had factor loadings of > 0.3 on the dominant factor except the 5 items: introductions, information confidentiality, and covered to respect privacy had loadings between 2 and 3, while family respected, had loadings between 1 and 2 (Table 3). The physical abuse item had the lowest loading below 0.1. Uniqueness for all items was < 0.9 except for these 5 items and the item knocked on door which also had a loading of 0.9. Cronbach’s alpha for the 26 items was 0.92. This did not change much with the sequential exclusion of the six items with low loadings and high uniqueness, with Cronbach’s alpha of 0.93 for the 20 items with high loadings and low uniqueness.

**Table 3:** Item loadings from factor analysis of 26 person-centered prenatal care-US scale items

	Loading on single factor		Loading on theoretical domains	
	Item loadings	Uniqueness	Item loadings	Uniqueness
Communication and Autonomy				
Introductions	0.29	0.92	0.33	0.89
Explain procedures	0.71	0.50	0.68	0.54
Consent obtained	0.52	0.73	0.51	0.74
Could ask questions	0.73	0.47	0.70	0.52
Encouraged to ask questions	0.51	0.74	0.58	0.66
Understanding checked	0.74	0.46	0.75	0.44
Questions answered	0.77	0.41	0.75	0.44
Felt heard	0.67	0.55	0.62	0.61
Involved in decisions	0.56	0.69	0.55	0.70
Dignity and Respect				
Treat you with respect	0.67	0.55	0.74	0.45
Family respected	0.15	0.98	0.16	0.97
Information confidential	0.27	0.93	0.18	0.97
Privacy: knocked	0.32	0.90	0.32	0.89
Privacy: covered	0.23	0.95	0.20	0.96
Verbal abuse	0.63	0.60	0.76	0.42
Physical abuse	0.08	1.00	0.13	0.98
Discrimination	0.40	0.84	0.46	0.79
Neglected	0.75	0.44	0.79	0.38
Experience valued	0.86	0.26	0.75	0.43
Responsive and Supportive care				
Time with provider	0.40	0.84	0.47	0.78
Showed they cared	0.79	0.37	0.73	0.46
Best care	0.77	0.41	0.72	0.48
Emotional wellbeing	0.67	0.55	0.74	0.46
Resources for emotional wellbeing	0.64	0.59	0.71	0.50
Trust	0.81	0.35	0.77	0.41
Safe	0.44	0.81	0.49	0.76

Factor analysis of the subset of items in each of the three conceptual domains also yielded one factor. All items loaded adequately (> 0.3) onto the first factor for the communication and autonomy and responsive and supportive care sub-domains (Table 3), and Cronbach’s alpha for both was ≥ 0.80 (Table 5). For the dignity and respect sub-domain, four items (family respected, information confidentiality, covered to respect privacy and physical abuse) had poor loadings between 1 and 3 (Table 3). Cronbach’s alpha for the 10 items in this sub-domain was 0.73 (increased to 0.81 when the four items with poor loadings were dropped).

Summative scores with all the scale items as well as with the reduced forms excluding items with poor loadings gave an average standardized score of 91 (Table 5). Subscale scores ranged from 87 for the responsive and supportive care sub-domain to 95 for the dignity and respect sub-domain. There were very strong correlations between the 26 and 20-item versions (*r* = 0.99) and between the different versions of the full scale and subscales (*r* > 0.7). All versions also showed moderately strong correlation with the PSQ and MORI scales (range of 0.37 to 0.64: Additional file 1: Appendix 3), suggesting good criterion validity based on our hypothesis.

#### PCMC-US scale

**Fig. 2:**
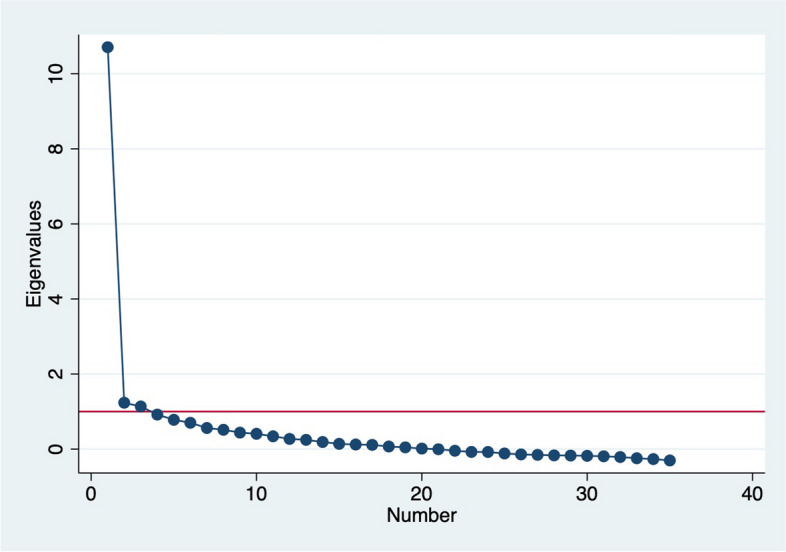
Scree plot from factor analysis of 35 person-centered maternity care-US scale items

There was good correlation between most items in the PCMC-US scale. The average inter-item correlation was 0.30, with most inter-item correlations between 0.2 and 0.7. Five items (introductions, baby’s feeding choice respected, pressured into decisions, physical abuse, and companionship) had inter-item correlations of less than 0.3 with most other items. The KMO values ranged from 0.58–0.94, with an overall KMO of 0.90. Factor analysis of the PCMC-US items also yielded one dominant factor with an eigenvalue of 10.7 accounting for 64% of the cumulative variance. This, together with two other factors with eigenvalues just above 1, accounted for 79% of the cumulative variance, but a decision was thus made to maintain the first factor based on the scree plot (Fig. 2). All items had factor loadings of $\ge$ 0.3 on the dominant factors except the six items: introductions, understandable language, baby’s feeding choice respected, pressured into decisions, and physical abuse, had loadings between 2 and 3, while companionship had a loading between 1 and 2 (Table 4). Uniqueness for all items was < 0.9 except for these 6 items and the item on information confidentiality which also had a loading of 0.91.

**Table 4:** Item loadings from factor analysis of 35 person-centered maternity care-US scale items

	Loading on single factor		Loading on theoretical domains	
	Item loadings	Uniqueness	Item loadings	Uniqueness
Communication and Autonomy				
Introductions	0.25	0.94	0.27	0.93
Felt heard	0.52	0.73	0.51	0.74
Involved in decisions	0.71	0.50	0.76	0.42
Explain procedures	0.63	0.61	0.76	0.42
Consent obtained	0.68	0.53	0.73	0.47
Understandable language	0.29	0.91	0.25	0.94
Felt informed	0.55	0.70	0.61	0.63
Could ask questions	0.71	0.49	0.72	0.48
Understanding checked	0.49	0.76	0.60	0.63
Birth position choice	0.38	0.86	0.31	0.91
Explain baby procedures	0.52	0.73	0.68	0.54
Birth preferences respected	0.58	0.66	0.59	0.66
Baby feeding choice respected	0.28	0.92	0.37	0.87
Pressured	0.25	0.93	0.31	0.90
Dignity and Respect				
Treat you with respect	0.70	0.50	0.73	0.47
Family respected	0.47	0.78	0.46	0.79
Information confidential	0.30	0.91	0.36	0.87
Privacy-covered	0.47	0.78	0.49	0.76
Verbal abuse	0.50	0.75	0.50	0.75
Physical abuse	0.25	0.94	0.22	0.95
Discrimination	0.39	0.85	0.22	0.95
Neglected	0.64	0.59	0.69	0.52
Experience valued	0.81	0.35	0.76	0.42
Customs respected	0.64	0.58	0.59	0.65
Responsive and Supportive care				
Emotional wellbeing	0.51	0.74	0.52	0.73
Pain management	0.67	0.55	0.74	0.45
Took best care	0.80	0.35	0.87	0.25
Trust	0.84	0.29	0.86	0.26
Safe	0.59	0.65	0.66	0.57
Companionship	0.16	0.97	0.21	0.95
Timely response	0.57	0.68	0.61	0.63
Believed about pain	0.70	0.51	0.73	0.47
Support for baby feeding	0.37	0.87	0.41	0.83
Comfortable birth environment	0.63	0.60	0.66	0.56
Wait time	0.48	0.77	0.51	0.74

The Cronbach’s alpha for the 35 items was 0.93. This did not change much with the sequential exclusion of items with low loadings, with Cronbach’s alpha of 0.94 for the 30 items with highest loadings (Table 5). Factor analysis of the subset of items in each of the three conceptual domains also yielded 1 factor. Most items loading adequately onto the first factor for all sub-domains, with 1 to 2 items having loadings of between 2 and 3 on each (Table 4). Cronbach alpha for all sub-domains >  = 0.79 (Table 5).

**Table 5:** Scale reliability and scores

	Internal Consistency			Standardized scores				
Scale component	*No. of items*	*Average inter-item correlation*	*Cronbach Alpha*	*N*	*Mean*	*Std. dev*	*Min*	*Max*
**PCPC-US scale**								
Full scale	26	0.31	0.92	315	91.33	12.16	28.21	100
Shorter version	20	0.41	0.93	315	91.32	14.03	16.67	100
Communication and autonomy	9	0.37	0.84	315	90.68	14.57	7.41	100
Dignity and respect	10	0.21	0.73	315	94.90	8.73	44.44	100
Responsive and supportive care	7	0.43	0.84	315	87.04	17.63	4.76	100
**PCMC-US scale**								
Full scale	35	0.30	0.94	286	90.57	12.19	19.61	100
Shorter version	30	0.35	0.94	286	91.43	12.92	18.39	100
Communication and autonomy	14	0.27	0.84	286	91.29	12.05	19.05	100
Dignity and respect	10	0.27	0.79	286	94.80	10.16	37.04	100
Responsive and supportive care	11	0.37	0.87	286	85.80	16.79	6.06	100

*Abbreviations: PCPC-US* person-centered prenatal care, *PCMC-US* person-centered maternity care

Summative scores with all the scale items as well as with the reduced forms excluding the five items with poor loadings give an average standardized score of > 90 (Table 5). Subscale scores range from 85 for the responsive and supportive care sub-domain to 95 for the dignity and respect sub-domain. There were very strong correlations between the different versions, with correlation coefficients of 0.99 between the 30 and 35 items versions of the full scale and > 0.80 with the subscales. All versions also show moderately strong correlation with the MORI scale scores (ranging from 0.62 to 0.70) (Additional file 1: Appendix 3), suggesting good criterion validity.

### Factors associated with PCMC-US and PCPC-US scale scores

In bivariate analysis (Additional file 1: Appendix 4 and 5) there were statistically significant differences in the PCPC or PCMC scores by race and ethnicity, birth country, interview language, education, housing instability, frequency of discrimination because of race or ethnicity, worry about being treated or judged unfairly because of race or ethnicity, Medi-Cal coverage, self-rated health, and the screening and delivery period. On average, Black participants had lower PCPC scores than Latinx participants. Participants who were born in Mexico had higher PCPC and PCMC scores than those who were born in the US while having ever been homeless was associated lower PCPC and PCMC scores. In addition, often experiencing discrimination because of race or ethnicity and worried about being treated or judged unfairly because of race or ethnicity were associated with lower PCPC scores. Completing the survey in Spanish and having Medi-Cal coverage were associated with higher PCMC scores as compared to completing it in English and not insured by Medi-Cal respectively, while college education or higher, receiving some prenatal care over phone, reporting good or fair/poor health status, was associated with lower PCMC scores than those with high school education or less, those who did not receive any care over phone, and those who reported very good self-rated health respectively. Finally, those who were screened or gave birth during the pre-vaccine period (before May 2021) had lower PCMC scores than those who were screened or gave birth later in the pandemic later.

The significant predictors in the final bootstrapped multivariate model were birth country, housing instability, worry about being treated or judged unfairly because of race or ethnicity, self-rated health, and delivery period. Women who were born in Mexico had higher PCMC scores compared to those born in the US (Table 6). In addition, those who reported having ever been homeless and worried about being treated or judged unfairly because of race or ethnicity had lower PCPC scores than those who reported never having been homeless and not worried about being treated or judged unfairly because of race or ethnicity respectively. Those who reported good or fair/poor health status had lower PCMC scores than those who reported very good self-rated health and those who gave birth later when COVID medications were available had higher PCMC scores than those who gave birth earlier in the pandemic. These differences provide evidence of known-groups validity.

**Table 6:** Multivariate analysis of the effect of predictors on PCMC-US and PCPC-US scale scores

	PCPC-US scale scores	PCMC-US scale scores
Predictors	B (95% CI) ^* †^	B (95% CI) ^* †^
Birth country		
United States	Reference	Reference
Mexico	1.17 (-1.49 – 3.84)	3.57** (1.24 – 5.90)
Other	1.71 (-3.57 – 6.99)	-2.07 (-10.2 – 6.10)
Ever been homeless		
No	Reference	Reference
Yes	-6.03* (-12.0 – -0.035)	-3.77 (-8.38 – 0.84)
Worry about being treated or judged unfairly because of race or ethnicity		
Never	Reference	Reference
Not very often	-0.76 (-4.08 – 2.55)	-1.24 (-4.82 – 2.34)
Somewhat often	-4.67* (-8.67 – -0.66)	-2.08 (-5.52 – 1.36)
Very often	1.54 (-3.38 – 6.47)	3.11 (-1.30 – 7.53)
How do you describe your general health?		
Excellent		Reference
Very good		-2.31 (-5.47 – 0.85)
Good		-4.46** (-7.17 – -1.76)
Fair or poor		-6.71* (-11.9 – -1.53)
Delivery Period		
COVID-19 Mass-Vaccine^‡^		Reference
COVID-19 Pre-Vaccine^§^		-6.11 (-21.4 9.19)
COVID-19 Medication^||^		2.66* (0.050 – 5.27)
Constant	92.6*** (90.9 – 94.3)	92.9*** (90.3 – 95.4)
N^¶^	308	278
R-squared	0.057	0.131
BIC	2439	2182.1

*Abbreviations: PCPC-US* person-centered prenatal care, *PCMC-US* person-centered maternity care, *CI* confidence intervals

^*^Data are reported as B (95% CI) unless otherwise indicated

^†^* *p* < 0.05 ** *p* < 0.01 *** *p* < 0.001

^‡^COVID-19 Mass-Vaccine dates include 05/01/21 – 02/28/22

^§^COVID-19 Pre-Vaccine dates include 03/16/20 – 04/30/21

^||^COVID-19 Medication dates include 03/01/22 – present

^¶^Lower Ns due to missing observations for some predictors

## Discussion

In this paper we describe the psychometric properties of the PCMC-US and PCPC-US scales in a low-income predominantly Latinx-identifying population in California. The findings provide support for the construct, criterion, and know-group validity, as well as the reliability, of the scales in this population. The average inter-item correlations were between 0.20 and 0.40, for both scales, which falls within the ideal range for items in a scale, suggesting that while the items are reasonably homogenous, they contain sufficiently unique variance to not be isomorphic with each other.

Although a few items have inter-item correlations and factor loadings below recommended cut-offs, exclusion on these items do not substantially change the Cronbach alpha values which are all well above the recommended levels of 0.7. Further, the 20 item-version of the PCPC-US scale and 30-item version of the PCMC-US scale, which include only items with the best fit, are strongly correlated with the full 26- and 35-item versions used in this analysis, suggesting these sub-set of items are adequate to capture the overall levels of PCPC-US and PCMC-US if a smaller sub-set of items is desired. Moderate correlations between the PSQ and MORI suggest that while they measure related constructs, they capture different domains. Finally, the scales can discriminate by groups that are likely to have different experiences, despite a relatively homogenous sample.

The findings of this study regarding validity and reliability of the scales in a sample of low-income predominantly Latinx-identifying participants are generally consistent with the original validation studies, which consisted of predominantly high-SES Black women [33, 34]. Specifically, the performance of the overall scales is similar to the original study in terms of high validity and reliability. There were, however, some differences in the item loadings: although all scale items loaded well in the in the initial validation analysis, a few items loaded poorly in this sample of Latinx-identifying participants. This is likely due to difference in the distribution of these items, potentially due to differences in the interpretation of the questions or differences in the actual care received.

The average scores across the studies are also similar: the PCPC score in the prior study was 91.8 (SD = 11.1) [33], compared to 92.01 (SD = 11.3) in the current study, while the PCMC score was 89.1 (SD = 14.0) [34], compared to 90.15 (SD = 12.93) in this study. The high scores are, however, surprising given that the study sample is a low-income minoritized population, whose experiences may be influenced by both racism and classism [16, 20, 51]. One possible reason for the high scores is that all participants were participating in an enhanced prenatal care program in which person-centered care is prioritized.

The analysis on factors associated with receipt of PCPC-US and PCMC-US are also consistent with prior studies with minoritized groups [17, 21, 22, 24, 52]. Even in this relatively homogenous group, the intersection of racism and classism is still reflected in the differences in scores based on immigration status (captured by birth country), SES (captured by unstable housing), and in worry about experiences of discrimination. The non-significant differences in other measures of SES such as having public or no insurance, employment, education, and income, in the final models, is likely due to lower variability in these measures in the low-income population, with ever having been homeless being the most discriminating factor. Similarly, little variability in racial and ethnic identities limits inferences on these, but the finding on discrimination reflects differences based on interpersonal experiences of racism [16, 53, 54].

The challenges of providing person-centered care during the early phase of the COVID-19 pandemic when several restrictions were put in place, including limiting birth companions, are also captured by the scales, reflected in the lower PCMC scores in the earlier phases of the pandemic [55, 56]. Other variables found to be associated with person-centered care in prior studies such as late start of prenatal care and lack of continuity of care and racial discordance with provider were not measured in the current study [52, 57–59].

Overall, the findings of our current study are consistent with existing literature regarding the characteristics of person-centered care for Latinx-identifying patients [60–63]. In a qualitative study of Latinx-identifying prenatal patients, Bergman and Connaughton reported 5 key themes of patient-centered care, including: a friendly relationship, effective medical care, spoken Spanish language, understanding of medical information, and elimination of racism [from the healthcare setting] [60]. They emphasized that training health care staff on the importance of displaying friendly communicative behaviors engenders greater trust in the healthcare team. Three additional studies had similar findings, reporting that cultural and linguistic competence were the most important factors informing person-centered care of Latinx-identifying patients [61–63]. To address the need for cultural competence during prenatal care, these authors conducted a follow up study of group prenatal care for Latinx-identifying pregnant people and found that patients participating in culturally competent group prenatal care experienced greater satisfaction and engagement with their care (e.g., more likely to establish a medical home for their child, and attend their postpartum appointments) [64]. Furthermore, multiple studies have found that addressing the need for linguistic competence includes the use of professional interpretation rather than ad hoc interpretation [21, 61, 65, 66]. Doing so improves patient trust and satisfaction [65]. Interestingly, in this study, completing the survey in Spanish was associated with higher PCMC scores in bivariate analysis. This may suggest that the more positive PCMC scores of Spanish-speaking participants reflect linguistically and culturally competent care. Feedback from our field staff also suggest that although language barrier influenced experiences of participants, the Spanish speaking foreign born participants tended to feel so grateful to have care that they were not bothered much by how staff treated them, which might explain the higher scores.

### Strengths and limitations

This is the first study to assess the psychometric properties of the PCMC-US and PCPC-US scales in a Latinx population. Theses scales were developed using a community-engaged approach embedded in standard instrument development methods. Starting with validated tools provided a rigorous, evidence and theory-based foundation. Expert reviews and cognitive interviews with people from racial and ethnic minoritized groups ensured content validity as well as relevance to the experiences of people in those groups. The current study provides additional evidence of construct, criterion, and discriminant validity in a Latinx population. A potential limitation is generalizability to other racial and ethnic groups and people of other SES, given this was a low-income Latinx population. However, given the scale performed similarly well in the previous validation in a high-income Black population, the findings suggest the scale will likely have similar levels of validity and reliability in diverse populations. Given the major need for cultural and linguistic adaptations in the care of Latinx patients, use of validated instruments such as the PCPC-US and PCMC-US scales provide valuable measures for interventions aimed at improving person-centered care and addressing existing inequities in obstetric and perinatal outcomes. Validations in other populations are however needed to ensure their appropriateness in other populations besides Black and Latinx individuals. Further, although the scales performed well in self-administered surveys in the previous validation in a predominantly high-SES Black population, the data collection in this study was only via interviewer-administered surveys. Thus, we are unable to speak directly to how well the scales will work as self-administered surveys in the low-SES predominantly Latinx population. Future studies should examine this.

Respondent burden due to the length of the scale also may be a limitation. Several items are needed given the multidimensional nature of person-centered care and the assessment of the relevance of the items included during the initial validation activities [33, 34]. However, given the high correlation between the 20- and 26-item versions of the PCPC-US and the 30- and 35-item versions of the PCMC-US scales, these shorter versions can be used where abbreviated scales are desired. While the longer scales may be more helpful for quality improvement efforts where the goal is to identify specific behaviors for improvement, the shorter scales can be used where only the summative score is needed. The sub-scales can also be used individually where necessary, although we recommend measuring all three domains to assess PCPC and PCMC in a holistic manner.

Finally, in our data the scale scores were highly left skewed, requiring the use of a non-parametric method (bootstrapping) in the multivariate analysis with the scores as the outcomes. Thus, when the scores are used as outcome variables in statistical analyses, the distribution of the scores should be examined. In situations where the distribution is highly non-normal appropriate statistical methods, such as nonparametric methods (e.g., bootstrapping or rank-based methods), should be used. Most statistical analyses, however, make no distributional assumptions about covariates. So, the left-skew should not be an issue if the scales are being used as covariates.

## Conclusions

The PCPC-US and PCMC-US scales, which were initially developed using a community-engaged process and found to have good psychometric properties in a largely high-income sample of Black women, were shown to also have good psychometric properties in a sample of largely low-income Latinx women. Given the increasing documentation of the poor experience of racial and ethnic minoritized groups in health care settings, it is important that these experiences are documented in a systematic manner. These two scales provide valid and reliable tools to measure person-centered care experiences among racial and ethnic minoritized groups during pregnancy and birth. These tools will also enable needs assessments to inform interventions as well as the evaluation of interventions to improve the experiences of racial and ethnic minoritized groups in health care settings during pregnancy and childbirth. In addition, having validated tools will enable assessment of changes across time and comparison across settings to drive as well as serve as an accountability tool in efforts to reduce the inequities in pregnancy and birth outcomes.

### Supplementary Information

**Additional file 1: Appendix 1a.** Distribution of PCPC items. **Appendix 1b.** Histogram of PCPC scores. **Appendix 2a.** Distribution of PCMC items. **Appendix 2b.** Histogram of PCMC scores. **Appendix 3.** Correlations between the different versions of the scales, sub-scales, and the MORI and PSQ scales. **Appendix 4.** Bivariate analysis of the effect of predictors on PCPC-US scores. **Appendix 5.** Bivariate analysis of the effect of predictors on PCMC-US scores.

## Data Availability

The data that support the findings of this study are available from the corresponding author upon reasonable request.
