# Supplementary material for: Psychometric assessment of the US person-centered prenatal and maternity care scales in a low-income predominantly Latinx population in California
Source: BMC Womens Health. 2023 Nov 17;23:616. doi: 10.1186/s12905-023-02721-5 (PMC10656820; doi:10.1186/s12905-023-02721-5)
Supplement: Supplementary file 1 — Additional file 1: Appendix 1a. Distribution of PCPC items. Appendix 1b. Histogram of PCPC scores. Appendix 2a. Distribution of PCMC items. Appendix 2b. Histogram of PCMC scores. Appendix 3. Correlations between the different versions of the scales, sub-scales, and the MORI and PSQ scales. Appendix 4. Bivariate analysis of the effect of predictors on PCPC-US scores. Appendix 5. Bivariate analysis of the effect of predictors on PCMC-US scores. [file 12905_2023_2721_MOESM1_ESM.docx]

| Appendix 1a Distribution of PCPC items | | |
| --- | --- | --- |
|  | No. | % |
| Introductions |  |  |
| No, none of them | 4 | 1.3 |
| Yes, a few of them | 43 | 13.7 |
| Yes, most of them | 52 | 16.5 |
| Yes, all of them | 216 | 68.6 |
| Total | 315 | 100 |
|  |  |  |
| Explain procedures |  |  |
| No, never | 5 | 1.6 |
| Yes, a few times | 10 | 3.2 |
| Yes, most of the time | 23 | 7.3 |
| Yes, all the time | 276 | 87.9 |
| Total | 314 | 100 |
|  |  |  |
| Consent obtained |  |  |
| No, never | 6 | 1.9 |
| Yes, a few times | 5 | 1.6 |
| Yes, most of the time | 15 | 4.8 |
| Yes, all the time | 287 | 91.7 |
| Total | 313 | 100 |
|  |  |  |
| Could ask questions |  |  |
| No, never | 6 | 1.9 |
| Yes, a few times | 5 | 1.6 |
| Yes, most of the time | 25 | 7.9 |
| Yes, all the time | 279 | 88.6 |
| Total | 315 | 100 |
|  |  |  |
| Encouraged to ask questions |  |  |
| No, never | 17 | 5.4 |
| Yes, a few times | 22 | 7 |
| Yes, most of the time | 27 | 8.6 |
| Yes, all the time | 249 | 79.1 |
| Total | 315 | 100 |
|  |  |  |
| Understanding checked |  |  |
| No, never | 7 | 2.2 |
| Yes, a few times | 19 | 6 |
| Yes, most of the time | 21 | 6.7 |
| Yes, all the time | 268 | 85.1 |
| Total | 315 | 100 |
|  |  |  |
| Questions answered |  |  |
| No, never | 5 | 1.6 |
| Yes, a few times | 16 | 5.1 |
| Yes, most of the time | 35 | 11.2 |
| Yes, all the time | 258 | 82.2 |
| Total | 314 | 100 |
|  |  |  |
| Felt heard |  |  |
| No, never | 18 | 5.7 |
| Yes, a few times | 18 | 5.7 |
| Yes, most of the time | 42 | 13.4 |
| Yes, all the time | 236 | 75.2 |
| Total | 314 | 100 |
|  |  |  |
| Involved in decisions |  |  |
| No, never | 6 | 1.9 |
| Yes, a few times | 15 | 4.8 |
| Yes, most of the time | 24 | 7.7 |
| Yes, all the time | 268 | 85.6 |
| Total | 313 | 100 |
|  |  |  |
| Treat you with respect |  |  |
| No, never | 1 | 0.3 |
| Yes, a few times | 5 | 1.6 |
| Yes, most of the time | 18 | 5.7 |
| Yes, all the time | 291 | 92.4 |
| Total | 315 | 100 |
|  |  |  |
| Family respected |  |  |
| No, never | 2 | 0.6 |
| Yes, a few times | 4 | 1.3 |
| Yes, most of the time | 93 | 29.6 |
| Yes, all the time | 215 | 68.5 |
| Total | 314 | 100 |
|  |  |  |
| Information confidential |  |  |
| No, never | 1 | 0.3 |
| Yes, a few times | 4 | 1.3 |
| Yes, most of the time | 11 | 3.5 |
| Yes, all the time | 297 | 94.9 |
| Total | 313 | 100 |
|  |  |  |
| Privacy: knocked |  |  |
| No, never | 8 | 2.5 |
| Yes, a few times | 8 | 2.5 |
| Yes, most of the time | 31 | 9.8 |
| Yes, all the time | 268 | 85.1 |
| Total | 315 | 100 |
|  |  |  |
| Privacy: covered |  |  |
| No, never | 5 | 1.6 |
| Yes, a few times | 5 | 1.6 |
| Yes, most of the time | 22 | 7 |
| Yes, all the time | 282 | 89.8 |
| Total | 314 | 100 |
|  |  |  |
| Verbal abuse |  |  |
| Yes, a few times | 10 | 3.2 |
| Yes, once | 5 | 1.6 |
| No, never | 300 | 95.2 |
| Total | 315 | 100 |
|  |  |  |
| Physical abuse |  |  |
| Yes, many times | 1 | 0.3 |
| Yes, once | 3 | 1 |
| No, never | 311 | 98.7 |
| Total | 315 | 100 |
|  |  |  |
| Discrimination |  |  |
| Yes, many times | 3 | 1 |
| Yes, a few times | 1 | 0.3 |
| Yes, once | 7 | 2.2 |
| No, never | 302 | 96.5 |
| Total | 313 | 100 |
|  |  |  |
| Neglected |  |  |
| Yes, many times | 7 | 2.2 |
| Yes, a few times | 12 | 3.8 |
| Yes, once | 13 | 4.1 |
| No, never | 282 | 89.8 |
| Total | 314 | 100 |
|  |  |  |
| Experience valued |  |  |
| No, never | 7 | 2.2 |
| Yes, a few times | 18 | 5.7 |
| Yes, most of the time | 37 | 11.8 |
| Yes, all the time | 253 | 80.3 |
| Total | 315 | 100 |
|  |  |  |
| Time with provider |  |  |
| It was extremely short | 10 | 3.2 |
| It was very short | 12 | 3.9 |
| It was somewhat short | 75 | 24.2 |
| It was just right | 213 | 68.7 |
| Total | 310 | 100 |
|  |  |  |
| Showed they cared |  |  |
| No, never | 9 | 2.9 |
| Yes, a few times | 22 | 7 |
| Yes, most of the time | 32 | 10.2 |
| Yes, all the time | 252 | 80 |
| Total | 315 | 100 |
|  |  |  |
| Best care |  |  |
| No, never | 10 | 3.2 |
| Yes, once | 17 | 5.4 |
| Yes, a few times | 43 | 13.7 |
| Yes, many times | 245 | 77.8 |
| Total | 315 | 100 |
|  |  |  |
| Emotional wellbeing |  |  |
| No, never | 24 | 7.6 |
| Yes, a few times | 33 | 10.5 |
| Yes, most of the time | 36 | 11.5 |
| Yes, all the time | 221 | 70.4 |
| Total | 314 | 100 |
|  |  |  |
| Resources for emotional wellbeing | |  |
| No, never | 31 | 9.9 |
| Yes, a few times | 29 | 9.2 |
| Yes, most of the time | 78 | 24.8 |
| Yes, all the time | 176 | 56.1 |
| Total | 314 | 100 |
|  |  |  |
| Trust |  |  |
| No, never | 3 | 1 |
| Yes, once | 16 | 5.1 |
| Yes, a few times | 32 | 10.2 |
| Yes, many times | 263 | 83.8 |
| Total | 314 | 100 |
|  |  |  |
| Safe |  |  |
| Yes, once | 4 | 1.3 |
| Yes, a few times | 28 | 8.9 |
| Yes, many times | 282 | 89.8 |
| Total | 314 | 100 |

Abbreviations: PCPC, person-centered prenatal care

Appendix 1b: Histogram of PCPC scores

| Appendix 2a Distribution of PCMC items | | | |
| --- | --- | --- | --- |
|  | No. | % |  |
| Introductions |  |  |  |
| No, none of them | 5 | 1.8 |  |
| Yes, a few of them | 19 | 6.6 |  |
| Yes, most of them | 40 | 14 |  |
| Yes, all of them | 222 | 77.6 |  |
| Total | 286 | 100 |  |
|  |  |  |  |
| Felt heard |  |  |  |
| No, never | 22 | 7.7 |  |
| Yes, a few times | 18 | 6.3 |  |
| Yes, most of the time | 26 | 9.1 |  |
| Yes, all the time | 220 | 76.9 |  |
| Total | 286 | 100 |  |
|  |  |  |  |
| Involved in decisions |  |  |  |
| No, never | 2 | 0.7 |  |
| Yes, a few times | 9 | 3.2 |  |
| Yes, most of the time | 20 | 7 |  |
| Yes, all the time | 254 | 89.1 |  |
| Total | 285 | 100 |  |
|  |  |  |  |
| Explain procedures |  |  |  |
| No, never | 2 | 0.7 |  |
| Yes, a few times | 6 | 2.11 |  |
| Yes, most of the time | 19 | 6.7 |  |
| Yes, all the time | 258 | 90.5 |  |
| Total | 285 | 100 |  |
|  |  |  |  |
| Consent obtained |  |  |  |
| No, never | 4 | 1.4 |  |
| Yes, a few times | 6 | 2.1 |  |
| Yes, most of the time | 24 | 8.5 |  |
| Yes, all the time | 248 | 87.9 |  |
| Total | 282 | 100 |  |
|  |  |  |  |
| Understandable language |  |  |  |
| No, never | 7 | 2.5 |  |
| Yes, a few times | 3 | 1.1 |  |
| Yes, most of the time | 24 | 8.4 |  |
| Yes, all the time | 251 | 88.1 |  |
| Total | 285 | 100 |  |
|  |  |  |  |
| Felt informed |  |  |  |
| No, never | 11 | 3.9 |  |
| Yes, a few times | 10 | 3.5 |  |
| Yes, most of the time | 25 | 8.7 |  |
| Yes, all the time | 240 | 83.9 |  |
| Total | 286 | 100 |  |
|  |  |  |  |
| Could ask questions |  |  |  |
| No, never | 9 | 3.2 |  |
| Yes, a few times | 5 | 1.8 |  |
| Yes, most of the time | 18 | 6.3 |  |
| Yes, all the time | 254 | 88.8 |  |
| Total | 286 | 100 |  |
|  |  |  |  |
| Understanding checked |  |  |  |
| No, never | 2 | 0.7 |  |
| Yes, a few times | 6 | 2.1 |  |
| Yes, most of the time | 32 | 11.2 |  |
| Yes, all the time | 246 | 86 |  |
| Total | 286 | 100 |  |
|  |  |  |  |
| Birth position choice |  |  |  |
| No | 40 | 14.2 |  |
| N/A | 46 | 16.3 |  |
| Yes | 196 | 69.5 |  |
| Total | 282 | 100 |  |
|  |  |  |  |
| Explain baby procedures |  |  |  |
| No, never | 3 | 1.1 |  |
| Yes, a few times | 7 | 2.5 |  |
| Yes, most of the time | 20 | 7 |  |
| Yes, all the time | 254 | 89.4 |  |
| Total | 284 | 100 |  |
|  |  |  |  |
| Birth preferences respected |  |  |  |
| No, never | 9 | 3.2 |  |
| Yes, a few times | 11 | 3.9 |  |
| Yes, most of the time | 36 | 12.6 |  |
| Yes, all the time | 229 | 80.4 |  |
| Total | 285 | 100 |  |
|  |  |  |  |
| Baby feeding choice respected |  |  |  |
| No, never | 14 | 4.9 |  |
| Yes, once | 8 | 2.8 |  |
| Yes, a few times | 22 | 7.8 |  |
| Yes, many times | 240 | 84.5 |  |
| Total | 284 | 100 |  |
|  |  |  |  |
| Pressured |  |  |  |
| Yes, all the time | 18 | 6.3 |  |
| Yes, most of the time | 7 | 2.5 |  |
| Yes, a few times | 16 | 5.6 |  |
| No, never | 243 | 85.6 |  |
| Total | 284 | 100 |  |
|  |  |  |  |
| Treat you with respect |  |  |  |
| Yes, a few times | 9 | 3.2 |  |
| Yes, most of the time | 39 | 13.6 |  |
| Yes, all the time | 238 | 83.2 |  |
| Total | 286 | 100 |  |
|  |  |  |  |
| Family respected |  |  |  |
| No, never | 2 | 0.7 |  |
| Yes, a few times | 5 | 1.8 |  |
| Yes, most of the time | 42 | 14.8 |  |
| Yes, all the time | 235 | 82.8 |  |
| Total | 284 | 100 |  |
|  |  |  |  |
| Information confidential |  |  |  |
| No, never | 4 | 1.4 |  |
| Yes, most of the time | 14 | 4.9 |  |
| Yes, all the time | 267 | 93.7 |  |
| Total | 285 | 100 |  |
|  |  |  |  |
| Privacy-covered |  |  |  |
| No, never | 8 | 2.8 |  |
| Yes, a few times | 6 | 2.1 |  |
| Yes, most of the time | 19 | 6.7 |  |
| Yes, all the time | 252 | 88.4 |  |
| Total | 285 | 100 |  |
|  |  |  |  |
| Verbal abuse |  |  |  |
| Yes, many times | 1 | 0.4 |  |
| Yes, a few times | 9 | 3.2 |  |
| Yes, once | 8 | 2.8 |  |
| No, never | 267 | 93.7 |  |
| Total | 285 | 100 |  |
|  |  |  |  |
| Physical abuse |  |  |  |
| Yes, a few times | 2 | 0.7 |  |
| Yes, once | 6 | 2.1 |  |
| No, never | 277 | 97.2 |  |
| Total | 285 | 100 |  |
|  |  |  |  |
| Discrimination |  |  |  |
| Yes, all the time | 1 | 0.4 |  |
| Yes, most of the time | 1 | 0.4 |  |
| Yes, a few times | 8 | 2.8 |  |
| No, never | 275 | 96.5 |  |
| Total | 285 | 100 |  |
|  |  |  |  |
| Neglected |  |  |  |
| Yes, many times | 8 | 2.8 |  |
| Yes, a few times | 20 | 7 |  |
| Yes, once | 6 | 2.1 |  |
| No, never | 252 | 88.1 |  |
| Total | 286 | 100 |  |
|  |  |  |  |
| Experience valued |  |  |  |
| No, never | 8 | 2.8 |  |
| Yes, a few times | 7 | 2.5 |  |
| Yes, most of the time | 24 | 8.5 |  |
| Yes, all the time | 245 | 86.3 |  |
| Total | 284 | 100 |  |
|  |  |  |  |
| Customs respected |  |  |  |
| No, never | 6 | 2.1 |  |
| Yes, a few times | 3 | 1.1 |  |
| Yes, most of the time | 37 | 13 |  |
| Yes, all the time | 239 | 83.9 |  |
| Total | 285 | 100 |  |
|  |  |  |  |
| Emotional wellbeing |  |  |  |
| No, never | 25 | 8.8 |  |
| Yes, a few times | 16 | 5.6 |  |
| Yes, most of the time | 33 | 11.6 |  |
| Yes, all the time | 210 | 73.9 |  |
| Total | 284 | 100 |  |
|  |  |  |  |
| Pain management |  |  |  |
| No, never | 10 | 3.5 |  |
| Yes, a few times | 9 | 3.2 |  |
| Yes, most of the time | 51 | 17.9 |  |
| Yes, all the time | 215 | 75.4 |  |
| Total | 285 | 100 |  |
|  |  |  |  |
| Took best care |  |  |  |
| No, never | 9 | 3.2 |  |
| Yes, a few times | 9 | 3.2 |  |
| Yes, most of the time | 42 | 14.7 |  |
| Yes, all the time | 226 | 79 |  |
| Total | 286 | 100 |  |
|  |  |  |  |
| Trust |  |  |  |
| No, never | 13 | 4.6 |  |
| Yes, a few times | 7 | 2.5 |  |
| Yes, most of the time | 27 | 9.4 |  |
| Yes, all the time | 239 | 83.6 |  |
| Total | 286 | 100 |  |
|  |  |  |  |
| Safe |  |  |  |
| No, never | 4 | 1.4 |  |
| Yes, a few times | 3 | 1.1 |  |
| Yes, most of the time | 23 | 8.1 |  |
| Yes, all the time | 255 | 89.5 |  |
| Total | 285 | 100 |  |
|  |  |  |  |
| Companionship |  |  |  |
| No, never | 109 | 38.4 |  |
| Yes, a few times | 7 | 2.5 |  |
| Yes, most of the time | 17 | 6 |  |
| Yes, all the time | 151 | 53.2 |  |
| Total | 284 | 100 |  |
|  |  |  |  |
| Timely response |  |  |  |
| No, never | 19 | 6.7 |  |
| Yes, a few times | 25 | 8.8 |  |
| Yes, most of the time | 33 | 11.6 |  |
| Yes, all the time | 208 | 73 |  |
| Total | 285 | 100 |  |
|  |  |  |  |
| Believed about pain |  |  |  |
| No, never | 5 | 1.8 |  |
| Yes, a few times | 22 | 7.8 |  |
| Yes, most of the time | 25 | 8.8 |  |
| Yes, all the time | 232 | 81.7 |  |
| Total | 284 | 100 |  |
|  |  |  |  |
| Support for baby feeding |  |  |  |
| No, never | 14 | 5 |  |
| Yes, a few times | 12 | 4.2 |  |
| Yes, most of the time | 22 | 7.8 |  |
| Yes, all the time | 235 | 83 |  |
| Total | 283 | 100 |  |
|  |  |  |  |
| Comfortable birth environment |  |  |  |
| No, never | 13 | 4.6 |  |
| Yes, a few times | 2 | 0.7 |  |
| Yes, most of the time | 23 | 8.2 |  |
| Yes, all the time | 244 | 86.5 |  |
| Total | 282 | 100 |  |
|  |  |  |  |
| Wait time |  |  |  |
| It was extremely short | 13 | 4.6 |  |
| It was very short | 12 | 4.2 |  |
| It was somewhat short | 84 | 29.5 |  |
| It was just right | 176 | 61.8 |  |
| Total | 285 | 100 |  |

Abbreviations: PCMC, person-centered maternity care

Appendix 2b: Histogram of PCMC scores

| Appendix 3 Correlations between the different versions of the scales, sub-scales, and the MORI and PSQ scales | | | | | | | | |
| --- | --- | --- | --- | --- | --- | --- | --- | --- |
| PCPC scale | | | | | | | |  |
|  | Full 26-item PCPC scale | Shorter 20-item version | Communi-cation and autonomy subscale | Dignity and respect subscale | Responsive and supportive care subscale | PSQ | MORI scale |  |
| Full 26-item PCPC scale | 1.00 |  |  |  |  |  |  |  |
| Shorter 20-item version | 0.99 | 1.00 |  |  |  |  |  |  |
| Communication and autonomy subscale | 0.94 | 0.93 | 1.00 |  |  |  |  |  |
| Dignity and respect subscale | 0.89 | 0.86 | 0.77 | 1.00 |  |  |  |  |
| Responsive and supportive care subscale | 0.93 | 0.93 | 0.80 | 0.75 | 1.00 |  |  |  |
| PSQ | 0.47 | 0.45 | 0.44 | 0.37 | 0.46 | 1.00 |  |  |
| MORI scale | 0.64 | 0.62 | 0.64 | 0.58 | 0.55 | 0.45 | 1.00 |  |
|  |  |  |  |  |  |  |  |  |
| PCMC scale |  |  |  |  |  |  |  |  |
|  | Full 26-item PCPC scale | Shorter 30-item version | Communi-cation and autonomy subscale | Dignity and respect subscale | Responsive and supportive care subscale | MORI scale |  |  |
| Full 35-item PCMC scale | 1.00 |  |  |  |  |  |  |  |
| Shorter 30-item version | 0.99 | 1.00 |  |  |  |  |  |  |
| Communication and autonomy subscale | 0.94 | 0.93 | 1.00 |  |  |  |  |  |
| Dignity and respect subscale | 0.91 | 0.92 | 0.83 | 1.00 |  |  |  |  |
| Responsive and supportive care subscale | 0.94 | 0.93 | 0.81 | 0.81 | 1.00 |  |  |  |
| MORI scale | 0.66 | 0.65 | 0.65 | 0.62 | 0.57 | 1.00 |  |  |

Abbreviations: MORI, Mothers On Respect Index; PSQ, Prenatal Care Satisfaction Questionnaire; PCPC, Person-Centered Prenatal Care; PCMC, Person-Centered Maternity Care.

| Appendix 4 Bivariate analysis of the effect of predictors on PCPC-US scores | | | | | | |
| --- | --- | --- | --- | --- | --- | --- |
| Predictors | N* | Mean±SD | | B* (95% CI) | |  |
| Age, years |  |  | |  | |  |
| < 18 | 7 | 93.9±5.7 | | Reference | |  |
| 18-24 | 101 | 91.2±11.3 | | -7.41 (-20.62 – 5.80) | |  |
| 25-34 | 165 | 91.2±13.4 | | -6.95 (-20.21 – 6.31) | |  |
| ≥ 35 | 38 | 90.9±10.2 | | -5.91 (-19.75 – 7.93) | |  |
|  |  |  | |  | |  |
| Relationship status |  |  | |  | |  |
| Married, living with partner | 251 | 91.2±11.8 | | Reference | |  |
| Partnered, not living together | 35 | 90.1±16.4 | | -1.28 (-6.33 – 3.77) | |  |
| Single | 29 | 94±8.7 | | 3.49 (-2.28 – 9.26) | |  |
|  |  |  | |  | |  |
| Parity |  |  | |  | |  |
| 0 | 81 | 91.1±11.6 | | Reference | |  |
| 1 | 84 | 91.9±14.7 | | 0.98 (-3.40 – 5.36) | |  |
| 2 | 67 | 90.9±10.7 | | -0.85 (-5.75 – 4.06) | |  |
| 3 | 50 | 91.9±10.0 | | -0.31 (-5.80 – 5.17) | |  |
| 4+ | 32 | 90.7±12.8 | | -0.44 (-6.79 – 5.92) | |  |
|  |  |  | |  | |  |
| Race or ethnic group |  |  | |  | |  |
| African American or Black | 15 | 85.9±16.1 | | Reference | |  |
| Asian or Pacific Islander | 11 | 95.6±5.5 | | 13.24* (2.25 – 24.24) | |  |
| Bi- or multi-racial/ethnic | 10 | 82.2±21.2 | | 1.01 (-10.06 – 12.08) | |  |
| Latina, Latinx, or Hispanic | 246 | 92.2±10.9 | | 7.16 (-0.62 – 14.93) | |  |
| None of the above ^†^ | 4 | 97.4±3.6 | | 13.41 (-1.49 – 28.31) | |  |
| White | 29 | 87.7±16.1 | | 4.54 (-4.34 – 13.43) | |  |
|  |  |  | |  | |  |
| Birth country |  |  | |  | |  |
| United States | 200 | 90.6±13.1 | | Reference | |  |
| Mexico | 101 | 92.4±10.3 | | 1.8 (-2.86 – 6.47) | |  |
| Other ‡ | 13 | 93.1±9.6 | | 3.91 (-4.73 – 12.55) | |  |
|  |  |  | |  | |  |
| Interview language |  |  | |  | |  |
| English | 233 | 91.2±12.5 | | Reference | |  |
| Spanish | 82 | 91.7±11.3 | | -3.39 (-8.35 – 1.57) | |  |
|  |  |  | |  | |  |
| Highest level of education attained |  |  | |  | |  |
| Less than high school diploma, high school graduate, or GED | 181 | 92.2±10.9 | | Reference | |  |
| Some college | 101 | 90.8±13.5 | | -1.91 (-5.64 – 1.82) | |  |
| College graduate or professional or graduate degree | 32 | 88.4±14.6 | | -6.53* (-12.11 – -0.95 ) | |  |
|  |  |  | |  | |  |
| Monthly household income, $ |  |  | |  | |  |
| < 1000 | 64 | 87.2±16.9 | | Reference | |  |
| 1000 – 2000 | 103 | 93.1±9.5 | | 5.56 (1.23 – 9.89) | |  |
| 2001 – 3000 | 76 | 92.9±9.1 | | 4.85* (0.18 – 9.51) | |  |
| > 3000 | 58 | 90.5±13.4 | | 2.98 (-2.19 – 8.15) | |  |
|  |  |  | |  | |  |
| Currently Employed |  |  | |  | |  |
| No | 204 | 90.9±12.5 | | Reference | |  |
| Yes | 111 | 92.2±11.5 | | 1.25 (-2.11 – 4.60) | |  |
|  |  |  | |  | |  |
| Medi-Cal Coverage |  |  | |  | |  |
| No | 19 | 89.3±14.4 | | Reference | |  |
| Yes | 296 | 91.5±12.0 | | 0.63 (-6.08 – 7.35) | |  |
|  |  |  | |  | |  |
| Ever been homeless |  |  | |  | |  |
| No | 267 | 92.1±10.5 | | Reference | |  |
| Yes | 42 | 85.9±19.3 | | -6.41* (-11.18 – -1.63) | |  |
|  |  |  | |  | |  |
| Have you ever tested positive for COVID-19? |  |  | |  | |  |
| No | 177 | 92.7±10.2 | | Reference | |  |
| Yes | 88 | 89.6±14.4 | | -0.79 (-4.33 – 2.75) | |  |
| Not asked | 50 | 89.6±13.7 | | -1.59 (-5.84 – 2.67) | |  |
|  |  |  | |  | |  |
| Diagnosis of diabetes or hypertension |  |  | |  | |  |
| No | 248 | 91.1±12.6 | | Reference | |  |
| Yes | 67 | 92.2±10.3 | | 1.93 (-1.74 – 5.61) | |  |
|  |  |  | |  | |  |
| Diagnosis of mental health condition |  |  | |  | |  |
| No | 240 | 92±11.0 | | Reference | |  |
| Yes | 75 | 89.2±15.1 | | 0.65 (-3.09 – 4.39) | |  |
|  |  |  | |  | |  |
| Prior preterm birth, low birth weight infant, or other composite outcome |  |  | |  | |  |
| No | 233 | 91.8±10.8 | | Reference | |  |
| Yes | 82 | 90.1±15.5 | | -0.46 (-4.01 – 3.10) | |  |
|  |  |  | |  | |  |
| How often do you feel discriminated against because of your race or ethnicity? |  |  | |  | |  |
| Never | 181 | 92.2±10.5 | | Reference | |  |
| Rarely | 59 | 91.9±12.0 | | 0.28 (-3.78 – 4.33) | |  |
| Sometimes | 63 | 90.0±13.5 | | -0.25 (-4.77 – 4.27) | |  |
| Often | 12 | 81.8±22.2 | | -7.38 (-15.6 – 0.86) | |  |
|  |  |  | |  | |  |
| How often do you worry that you are treated or judged unfairly because of your race or ethnicity? |  |  | |  | |  |
| Never | 164 | 92.4±11.4 | | Reference | |  |
| Not very often | 67 | 91.4±12.5 | | -1.56 (-5.62 – 2.50) | |  |
| Somewhat often | 65 | 88.0±14.2 | | -4.35* (-8.61 – -0.09) | |  |
| Very often | 19 | 93.2±8.2 | | 4.98 (-2.18 – 12.14) | |  |
|  |  |  | |  | |  |
| Received any care by phone or video |  |  | |  | |  |
| No | 218 | 91.3±12.8 | | Reference | |  |
| Yes | 97 | 91.5±10.6 | | 1.73 (-1.68 – 5.13) | |  |
|  |  |  | |  | |  |
| Screening Period |  |  | |  | |  |
| COVID-19 Mass-Vaccine ^§^ | 114 | 92.5±10.0 | | Reference | |  |
| COVID-19 Pre-Vaccine ^\|\|^ | 81 | 91.1±12.5 | | -1.44 (-4.91 – 2.04) | |  |
| COVID-19 Medication ^¶^ | 120 | 90.3±13.7 | | -2.18 (-5.31 – 0.94) | |  |
|  |  |  | |  | |  |
| Delivery Period |  |  | |  | |  |
| COVID-19 Mass-Vaccine ^§^ | 119 | 92.4±10.1 | | Reference | |  |
| COVID-19 Pre-Vaccine ^\|\|^ | 11 | 87.6±18.8 | | -4.82(-12.3,2.64) | |  |
| COVID-19 Medication ^¶^ | 148 | 92.0±11.1 | | -0.42(-3.33,2.50) | |  |
| No Delivery data | 37 | 86.1±17.9 | | -6.31**(-10.8,-1.85) | |  |
| Abbreviations: PCPC, person-centered prenatal care; CI, confidence intervals; GED, general education development certificate; COVID-19, Coronavirus disease -19 | | | | | | |
| * Lower Ns due to missing observations for some predictors. Statistical significance indicated as follows: * p<0.05 ** p<0.01 *** p<0.001 | |  |  | |  | |
| ^†^ Data include Native American, American Indian, and Alaska Native. | | |  | |  | |
| ‡ Data include El Salvador, Guatemala, Honduras, Nicaragua, Philippines, Armenia, Egypt, India. | | | | | | |
| ^§^ COVID-19 Mass-Vaccine dates include 05/01/21 – 02/28/22. | |  |  | |  | |
| ^\|\|^ COVID-19 Pre-Vaccine dates include 03/16/20 – 04/30/21. | |  |  | |  | |
| ^¶^ COVID-19 Medication dates include 03/01/22 – present. | |  |  | |  | |

| Appendix 5 Bivariate analysis of the effect of predictors on PCMC-US scores | | | | | | | | | | |  |
| --- | --- | --- | --- | --- | --- | --- | --- | --- | --- | --- | --- |
| Predictors | | N* | | | Mean±SD | | | B* (95% CI) | | |  |
| Age, years | |  | | |  | | |  | | |  |
| < 18 | | 4 | | | 92.1±7.6 | | | Reference | | |  |
| 18-24 | | 99 | | | 89.6±13.4 | | | 24.08 (3.01 – 51.16) | | |  |
| 25-34 | | 149 | | | 91.0±11.4 | | | 25.23 (-1.52 – 51.99) | | |  |
| ≥ 35 | | 30 | | | 90.6±13.6 | | | 28.03* (0.78 – 55.27) | | |  |
|  | |  | | |  | | |  | | |  |
| Relationship status | |  | | |  | | |  | | |  |
| Married, living with partner | | 225 | | | 90.9±12.1 | | | Reference | | |  |
| Partnered, not living together | | 35 | | | 88.8±11.9 | | | -0.38 (-5.33 – 4.77) | | |  |
| Single | | 26 | | | 90.3±13.9 | | | 3.03 (-3.43 – 9.49) | | |  |
|  | |  | | |  | | |  | | |  |
| Parity | |  | | |  | | |  | | |  |
| 0 | | 78 | | | 89.2±11.9 | | | Reference | | |  |
| 1 | | 72 | | | 91.1±11.8 | | | 0.06 (-4.67 – 4.78) | | |  |
| 2 | | 63 | | | 91.1±13.7 | | | -0.53 (-5.78 –4.77) | | |  |
| 3 | | 44 | | | 92.2±10.9 | | | 0.72 (-5.42 – 6.85) | | |  |
| 4+ | | 28 | | | 89.1±12.9 | | | 0.78 (-6.26 – 7.82) | | |  |
|  | |  | | |  | | |  | | |  |
| Race or ethnic group | |  | | |  | | |  | | |  |
| African American or Black | | 13 | | | 85.9±14.0 | | | Reference | | |  |
| Asian or Pacific Islander | | 9 | | | 92.0±6.8 | | | 9.56 (-2.58 – 21.69) | | |  |
| Bi- or multi-racial/ethnic | | 10 | | | 91.1±11.8 | | | 17.7* (-4.36 – 31.05) | | |  |
| Latina, Latinx, or Hispanic | | 226 | | | 91.7±10.9 | | | 8.53 (-0.54 – 17.59) | | |  |
| None of the above ^†^ | | 3 | | | 81.4±28.2 | | | -3.4 (-20.36 – 13.55) | | |  |
| White | | 25 | | | 83.6±18.2 | | | 0.7 (-9.36 – 10.77) | | |  |
|  | |  | | |  | | |  | | |  |
| Birth country | |  | | |  | | |  | | |  |
| United States | | 183 | | | 89.0±13.4 | | | Reference | | |  |
| Mexico | | 91 | | | 94.1±7.9 | | | 0.18 (-4.90 – 5.26) | | |  |
| Other ‡ | | 12 | | | 87.3±15.4 | | | -2.34 (-11.26 – 6.58) | | |  |
|  | |  | | |  | | |  | | |  |
| Interview language | |  | | |  | | |  | | |  |
| English | | 211 | | | 89.1±13.2 | | | Reference | | |  |
| Spanish | | 75 | | | 94.6±7.7 | | | 2.15 (-3.79 – 6.84) | | |  |
|  | |  | | |  | | |  | | |  |
| Highest level of education attained | |  | | |  | | |  | | |  |
| Less than high school diploma, high school graduate, or GED | | 161 | | | 92.4±9.3 | | | Reference | | |  |
| Some college | | 96 | | | 88.7±13.9 | | | -1.79 (-5.72 – 2.15) | | |  |
| College graduate or professional or graduate degree | | 28 | | | 86.6±18.0 | | | -2.13 (-9.71 – 3.89) | | |  |
|  | |  | | |  | | |  | | |  |
| Monthly household income, $ | |  | | |  | | |  | | |  |
| < 1000 | | 54 | | | 92±9.4 | | | Reference | | |  |
| 1000 – 2000 | | 98 | | | 91.6±13.1 | | | -2.34 (-7.17 – 2.49) | | |  |
| 2001 – 3000 | | 73 | | | 90.3±11.1 | | | -2.20 (-7.23 – 2.83) | | |  |
| > 3000 | | 49 | | | 87.8±14.4 | | | -2.17 (-7.87 – 3.54) | | |  |
|  | |  | | |  | | |  | | |  |
| Currently Employed | |  | | |  | | |  | | |  |
| No | | 182 | | | 90.5±13.3 | | | Reference | | |  |
| Yes | | 103 | | | 90.7±10.0 | | | 1.85 (-2.51 – 4.74) | | |  |
|  | |  | | |  | | |  | | |  |
| Medi-Cal Coverage | |  | | |  | | |  | | |  |
| No | | 18 | | | 84.6±17.0 | | | Reference | | |  |
| Yes | | 268 | | | 91±11.7 | | | 4.75 (-2.05 – 11.55) | | |  |
|  | |  | | |  | | |  | | |  |
| Ever been homeless | |  | | |  | | |  | | |  |
| No | | 240 | | | 91.5±11.0 | | | Reference | | |  |
| Yes | | 39 | | | 87.2±15.0 | | | -6.11* (-11.43 – -0.79) | | |  |
|  | |  | | |  | | |  | | |  |
| Have you ever tested positive for COVID-19? | |  | | |  | | |  | | |  |
| No | | 168 | | | 92.0±11.1 | | | Reference | | |  |
| Yes | | 67 | | | 89.5±10.2 | | | -1.73 (-5.60 – 2.14) | | |  |
| Not asked | | 51 | | | 87.2±16.7 | | | -3.85 (-8.15 – 0.46) | | |  |
|  | |  | | |  | | |  | | |  |
| Diagnosis of diabetes or hypertension | |  | | |  | | |  | | |  |
| No | | 229 | | | 90.9±11.4 | | | Reference | | |  |
| Yes | | 57 | | | 89.4±14.9 | | | -2.64 (-6.62 – 1.35) | | |  |
|  | |  | | |  | | |  | | |  |
| Diagnosis of mental health condition | |  | | |  | | |  | | |  |
| No | | 222 | | | 91.2±11.8 | | | Reference | | |  |
| Yes | | 64 | | | 88.4±13.3 | | | 0.28 (-3.73 – 4.29) | | |  |
|  | |  | | |  | | |  | | |  |
| Prior preterm birth, low birth weight infant, or other pregnancy complication | |  | | |  | | |  | | |  |
| No | | 207 | | | 91.1±11.6 | | | Reference | | |  |
| Yes | | 79 | | | 89.2±13.6 | | | 0.09 (-3.88 – 4.05) | | |  |
|  | |  | | |  | | |  | | |  |
| How would you describe your general health? | |  | | |  | | |  | | |  |
| Excellent | | 72 | | | 94.9±5.7 | | | Reference | | |  |
| Very good | | 69 | | | 90.5±11.8 | | | -2.71 (-7.21 – 1.79) | | |  |
| Good | | 103 | | | 89.7±11.7 | | | -6.75** (-10.72 – -2.79) | | |  |
| Fair/Poor | | 41 | | | 86±18.7 | | | -7.72** (-13.12 – -2.31) | | |  |
|  | |  | | |  | | |  | | |  |
| How often do you feel discriminated against because of your race or ethnicity? | |  | | |  | | |  | | |  |
| Never | 167 | | | 91.4±11.2 | | | Reference | | |  |  |
| Rarely | 54 | | | 91.1±10.8 | | | 0.13 (-4.17 – 4.44) | | |  |  |
| Sometimes | 56 | | | 88.5±15.1 | | | -3.08 (-7.91 – 1.75) | | |  |  |
| Often | 9 | | | 85.7±17.0 | | | -12.24* (-22.06 – -2.41) | | |  |  |
|  | |  | | |  | | |  | | |  |
| How often do you worry that you are treated or judged unfairly because of your race or ethnicity? | |  | | |  | | |  | | |  |
| Never | | 152 | | | 91.7±12.0 | | | Reference | | |  |
| Not very often | | 59 | | | 88.7±14.2 | | | 0.16 (-4.23 – 4.54) | | |  |
| Somewhat often | | 57 | | | 88.8±11.0 | | | 1.99 (-2.71 – 6.69) | | |  |
| Very often | | 18 | | | 92.8±9.3 | | | 5.82 (-1.57 – 13.22) | | |  |
|  | |  | | |  | | |  | | |  |
| Received any care by phone or video | |  | | |  | | |  | | |  |
| No | | 169 | | | 91.7±10.6 | | | Reference | | |  |
| Yes | | 90 | | | 88.5±14.3 | | | -1.62 (-5.11 – 1.88) | | |  |
|  | |  | | |  | | |  | | |  |
| Screening Period | |  | | |  | | |  | | |  |
| COVID-19 Mass-Vaccine ^§^ | | 128 | | | 91.6±11.1 | | | Reference | | |  |
| COVID-19 Pre-Vaccine ^\|\|^ | | 84 | | | 87.0±16.2 | | | -4.55** (-7.87 – -1.23) | | |  |
| COVID-19 Medication ^¶^ | | 74 | | | 92.9±6.8 | | | 1.34 (-2.11 – 4.79) | | |  |
|  | |  | | |  | | |  | | |  |
| Delivery Period | |  | | |  | | |  | | |  |
| COVID-19 Mass-Vaccine ^§^ | | 120 | | | 89.3±13.2 | | | Reference | | |  |
| COVID-19 Pre-Vaccine ^\|\|^ | | 9 | | | 79.3±27.4 | | | -10.1** (-17.1,-2.99) | | |  |
| COVID-19 Medication ^¶^ | | 132 | | | 92.6±8.1 | | | 3.29* (0.46,6.12) | | |  |
| Abbreviations: PCMC, person-centered maternity care; CI, confidence intervals; GED, general education development certificate; COVID-19, Coronavirus disease -19 | | | | | | | | | | | |
| * Lower Ns due to missing observations for some predictors. Statistical significance indicated as follows: * p<0.05 ** p<0.01 *** p<0.001 | | | | | | | | | | | |
| ^†^ Data include Native American, American Indian, and Alaska Native. | | | | | |  | | |  | | |
| ‡ Data include El Salvador, Guatemala, Honduras, Nicaragua, Philippines, Armenia, Egypt, India. | | | | | | | | | | | |
| ^§^ COVID-19 Mass-Vaccine dates include 05/01/21 – 02/28/22. | | | | | |  | | |  | | |
| ^\|\|^ COVID-19 Pre-Vaccine dates include 03/16/20 – 04/30/21. | | | | | |  | | |  | | |
| ^¶^ COVID-19 Medication dates include 03/01/22 – present. | | |  | | |  | | |  | | |
